# The Effects of Heat Stress on Sheep Welfare during Live Export Voyages from Australia to the Middle East

**DOI:** 10.3390/ani10040694

**Published:** 2020-04-16

**Authors:** Francesca Carnovale, Clive J. C. Phillips

**Affiliations:** 1 Centre for Animal Welfare and Ethics, School of Veterinary Science, The University of Queensland, Gatton, QLD 4343, Australia; francesca.carnovale@student.emu.ee; 2 Institute of Veterinary Medicine and Animal Sciences, Estonian University of Life Sciences, Kreutzwaldi 1, 51014 Tartu, Estonia

**Keywords:** heat stress, live export, mortality, sheep, temperature

## Abstract

**Simple Summary:**

Sheep are regularly exported from Australia to the Middle East, which is one of the world’s longest sea journeys. There is a particular risk to animal welfare in voyages departing Australia in the Southern Hemisphere winter and arriving in the Persian Gulf in the Middle East after about 15 days, into the Northern Hemisphere summer, because of the rapid transition from cold to hot temperatures. The threshold temperature when welfare problems occur is not well understood. We utilized data, including temperature and the number of sheep that died, collected on 14 shipments of sheep, travelling from Australia to the Middle East in the May to December period, between 2016 and 2018. Our modelling of the data suggested that sheep would experience heat stress in 50% of voyages between July and September offloading sheep at two of four Persian Gulf ports. Furthermore, when sheep were taken off the ship at Doha, the hottest port, first, the number dying on the ship increased. The results confirm the beneficial impact on animal welfare of restricting any sailing with a cargo of sheep from Australia to the Middle East in the Southern Hemisphere winter.

**Abstract:**

One of the world’s longest sea transport routes of live sheep for slaughter is from Australia to the Middle East. Heat stress is a major cause of mortality in shipments of sheep, particularly in sheep leaving Australia in the Southern Hemisphere winter to arrive in the Middle Eastern summer. Temperature and mortality data were utilized and recorded from fourteen voyages from Australia to the Middle East in May to December, 2016–2018, with the aim of determining when the welfare of the sheep began to be affected by elevated temperatures. Increases in heat stress were recorded at temperatures normally experienced in 50% of voyages between July and September offloading sheep at two of the four Persian Gulf ports, Doha and Dubai; however, small increases in recorded heat stress were not sufficient to increase mortality. Temperatures increased most rapidly when sheep were offloaded initially at Doha first, followed by other Gulf ports, and this resulted in higher mortality than when sheep were offloaded at other ports first. These results confirm benefits of restricting voyages leaving Australia in the Southern Hemisphere winter and suggest that shipments offloading at multiple ports should not offload at the hottest port, Doha, first.

## 1. Introduction

Live transport of cattle and sheep is increasing, due to growing demand for meat, centralisation of slaughterhouses with improved facilities, and the need for religious slaughter (e.g., for freshly slaughtered meat). Australia, with the second largest sheep population in the world, exports sheep worldwide, approximately one to two million annually, 98% of which go to the Middle East [1] (Table 1).

In 2018, of 1,259,860 live sheep that were loaded to be exported from Australia, 6629 (0.53%) died [1]. Long distance travel has become increasingly contentious, especially in the last few years, when reduced tariffs and greater availability of specialised ships have had global impact [3]. Mortality during live sheep export has been cited as a top welfare impact for the Australian sheep industry by stakeholders [4]. However, it is acknowledged that many sheep may suffer considerable distress without necessarily dying on shipments. Other welfare concerns that have been recognized include irritation of eye, nose and mouth mucosal surfaces because of ammonia [5], loss of balance and fatigue as a result of ship motion [6] and stress during exposure to high temperatures [7]. Compared with the sheep export in the Southern Hemisphere summer or autumn, shipments leaving Australia in the Southern Hemisphere winter or spring have higher mortality rates [8]. During these voyages, sheep are expected to be exposed to heat stress if the temperatures experienced after shipments cross equatorial regions and arrive in the Middle East are above their upper critical temperature, particularly if there is little circadian variation in temperature [9,10]. The extent of temporal variation is unclear, but a recent study on three vessels travelling from Australia to the Middle East suggests that periods of extreme heat may be limited to short periods of the day [11]. So far, the detrimental effects of heat stress on sheep during live export have mainly been investigated in climate control rooms. Little has been published about the importance of heat stress for sheep mortality on shipments under actual trade conditions. It is believed to be a major cause of sheep mortality during live export [12], particularly if shipments leave Australia in the Southern Hemisphere winter, bound for the Middle East.

The animals begin their journey in mild-cold temperatures (average minimum in SW Australia of 7 °C) in the Southern Hemisphere Australian winter, arriving after about twenty days in the Northern Hemisphere summer period of the Middle East, where temperatures above 40 °C dry bulb temperature may be experienced [13]. Forced ventilation is used to cool the sheep, although some ships have open decks above sea level, which expose sheep at the sides of the decks to natural ventilation, but also potentially to radiant heat from the sun. Besides the large amount of heat generated by solar radiation on the top and sides of the vessel, high temperatures occur beside the engine block, boiler room and heated fuel tank, as well as on sheep decks, where the high stocking density increases temperatures above ambient levels. Sheep heat output is a function of the heat released by each animal and the stocking density [14]. Temperatures on board are monitored on each deck and on the bridge, the latter being expected to be largely unaffected by heat generated by the cargo.

When the ships enter the Persian Gulf, after about 15 days, heat stress may ensue and welfare may be impaired if ambient temperatures exceed a threshold value for the particular type of animal, for example 28 °C Wet Bulb Temperature (T_WB_) for a 56 kg shorn adult Merino wether in moderate body condition score and acclimatized to winter temperatures at departure [7]. This is assumed to represent a 5% probability that such sheep would experience heat stress on any particular voyage. The mean 98th percentile values for average T_WB_ at Persian Gulf ports in July, August, and September are all above this threshold value (31.8 °C in Kuwait, 32.2 °C in Qatar, Doha, 31.5 °C in United Arab Emirates, Dubai, and 29.8 °C in Oman, Muscat [15]).

In order to predict the heat-stress-related mortality risk, a Heat Stress Risk Assessment model (HSRA) is included in the Australian Standards for the Export of Livestock [16]. The model uses wet bulb temperature, T_WB_, a more accurate measure of the likelihood of heat stress in sheep than dry bulb temperature, T_DB_, to compare with a pre-determined heat stress threshold (HST) for each category of sheep involved, with a reduction in stocking density if there is determined to be a high risk (>2%, CI 95%) of heat stress [17]. Sheep are tolerant to only a narrow band of T_WB_, so accurate estimation of heat stress is important [14,18,19]. However, the relationship between stocking density adjustment and heat stress relief has not been published for long haul sea transport and is especially needed for the voyages to the Middle East in the Northern Hemisphere summer [20]. Also, the HST and mortality limit (ML, the T_WB_ above which merino lambs or adults die) for the sheep category used by the HSRA model have never been formally published for shipments exporting sheep from Australia to the Middle East. 

The research that we have considered so far addresses only the risk of heat stress to sheep posed by the environmental conditions in which the ship travels, and in particular the Persian Gulf. However, given that climatic and other relevant data and mortality rates for each deck are collected daily on the ships, it is theoretically possible to examine the local environmental factors influencing mortality rate, in order to determine the validity of the sheep industry’s Heat Stress Risk Assessment Model. The need for this was highlighted when the Australian Government published its Technical Review Panel’s report into approaches to HSRA [7]. This panel aimed to support the industry to transition from a mortality-based risk assessment to one based on welfare impairment. The impact of the Technical Review Panel’s report is that there will be a blackout period when many winter shipments will not sail.

We analysed heat stress-related data from voyages from Australia to Middle East, with the objective of determining critical thresholds that would avoid serious welfare outcomes at high temperatures, thereby facilitating improved control of the heat stress risk.

## 2. Materials and Methods

### 2.1. Description of the Ships and Voyages Transporting Live Animals to the Middle East

Records of data obtained during 14 livestock voyages were provided by the then Department of Agriculture and Water Resources in the Australian federal government (Appendix Table A1). The mean number of sheep/voyage was 46,501 (SD 25461), with a range of 4466 to 77,988 head. These voyages took place between 2016 and 2018, from the ports of Fremantle and Adelaide in Australia to the ports of Kuwait, Eilat, Aqaba, Doha, Muscat and Dubai in the Persian Gulf and Red Sea. Two voyages had only one destination (Kuwait and Muscat); two had two destinations (Muscat and Kuwait; Eilat and Aqaba); six had three destinations (Doha, Kuwait and Dubai, *n* = 5; Doha, Kuwait and Muscat, *n* = 1). Three of these were in the following order: first stop Doha, second Kuwait and finally Dubai; two were in the order Kuwait, then Doha and finally Dubai; one was in the order Doha, then Kuwait and finally Muscat. The other four voyages had four destinations, Dubai, Kuwait, Doha and Muscat. Only two had the same order: first stop Dubai, second stop Kuwait, third Doha and finally Muscat; one had first stop Doha, second stop Kuwait, third stop Dubai and finally Muscat; one had first stop Kuwait, second Doha, third Dubai and finally Muscat. Voyages had a mean duration of 22.36 ± 4.13 d, range 13 to 30 d, with the first port destination being reached on average after 16.3 ± 2.8 d. Nine voyages started during the Australian winter: four during May, two in June, two in August and one in July. Of the other five, three started in September and two in November.

The vessels used were built for transporting unpackaged bulk cargo, and specialized for transport of live animals, with typical dimensions 180 × 31 m and an average speed of 12.0 kn. Nine voyages were on ships with ten decks, and five with nine. All decks were closed to the outside environment and were artificially ventilated at 20–30 air changes/h. The ship with nine decks had each deck split into two horizontally to double the capacity for sheep.

The gross tonnage of the ships was approximately 40,000 tons. The pen capacity of each ship was a mean of 23,267 m^2^, SD 2404, with each deck having a capacity of at least of 2000 m^2^. Sheep were enclosed in solid metal floored pens on each deck, each required by Australian law to be 2–4.5 m wide and length 1–2 × the width [20]. Each ship contained a mean of 3826 ± 2812.5 head per deck with a maximum of 11,226/deck, in total increasing the weight of the ship by on average 3433 ± 305 tonnes/ship. Sheep were loaded to a stocking density prescribed by the Australian Standard for the Export of Livestock [16] (approximately 0.35 m^2^/head).

Young and adult sheep were fed at 3% and 2% of their live weight per head per day, respectively, and fresh water supplied at 4–6 L /head/day, the higher amount if the ambient temperature exceeded 35 °C [16]. Water was produced by reverse osmosis of sea water during the voyages. The feed used was pelleted and had the following nutritional specification: moisture content 12%, and as a % of dry matter, ash 13%, crude protein between 9% and 12%, urea 1.2%, acid detergent fibre 18%–35% and metabolisable energy 8.0 MJ/kg dry matter or above. Feed was loaded in relation to the animals’ body weight [14] and stored in either large fodder tanks in the hull or in bulker bags on the roof. From the tanks, it was pumped by mechanical augers twice daily to storage bins on the roof, then delivered to the individual feed troughs, or pumped directly to troughs. The correct amount to auger to the tanks was determined by a feed manager. The feed was then supplied to the troughs at a point outlet and distributed along the lengths of the troughs by the crew but may have spilled onto the floor if no-one was in attendance. Bulker bags were distributed by individual crew members to the troughs. Left over feed was tipped into the pens on a daily basis. Additional feed was provided if sheep were thought to be losing weight, typically a third feed in the day. No bedding was provided in all but one voyage (on one sawdust was provided), and the excreta fell onto a solid floor. There it was, under normal conditions, rapidly dried by the high ventilation rate, forming a dry, friable powder on which the sheep lay.

Sheep mortalities on each deck and vessel cumulative mortalities were recorded by the ship veterinarian and reported to the captain daily. Wet bulb temperature (T_WB_ °C), dry bulb temperature (T_DB_ °C), and relative humidity (RH, in %) were measured daily on individual decks by wet and dry bulb mercury thermometers (e.g., Camon Automatic Instruments, Beijing, PR China), above sheep height to avoid damage, and on the bridge of the ship. Sea temperature was recorded daily by electronic sensors in the ships’ seawater intake ports (used for engine cooling), relayed to the engine room. Feed wet matter intake and water allocation and consumption were also recorded daily on a deck basis. Heat stress characteristics were also subjectively recorded by the veterinarian on a daily basis: using a numerical scale of 1 to 3:1 normal, 2 moderate and 3 severe). Scores entered as 1/2 (1 or 2) and 2/3 (2 or 3) were entered as 1.5 and 2.5, respectively. The scale used by ship veterinarians was determined using sheep behaviour and posture indicators (Table 2).

### 2.2. Statistical Analysis

Statistical analysis was performed using Minitab 18.0 (Minitab Version 18; Minitab Inc., State College, PA, USA). A general linear model (GLM) with stepwise regression was used to determine which environmental variables and relevant interactions (discrete variable: heat stress; continuous variables: month and day of voyages, number of animals loaded, temperatures on the bridge and each deck, feed and water consumption) were statistically related to mortality rate, measured on each deck in % of those present/day. Sea temperature and a temperature humidity index [21] were initially included in the models but were discarded because of auto collinearity with other temperature-related variables, as determined by inspection of variance inflation factors. Residuals were confirmed to be normally distributed by plotting their distribution graphically.

Scatterplots were generated for the daily vessel mortality rate (%), T_WB_ on sheep decks (°C), bridge T_WB_ (°C), sheep feed intake (kg) and water consumption (L) for each day of the fourteen voyages. Three-dimensional graphs were developed to represent the relationships with deck mortality rate. Non-linear regression was used to plot relationships for variables identified as significantly (*p* < 0.05) related to mortality rate. Following inspection of scatterplots, we used a power function to relate day of voyage, and an exponential function to relate mortality, to deck and bridge T_WB_ °C. Differences in daily deck mortality rate for heat stress scores were tested with Fisher’s pairwise comparisons.

An ordinal logistic regression model with a Logit function was developed to examine the relationships between the variable heat stress score with environment, health and hygiene variables and the voyage characteristics.

## 3. Results

Mean, standard deviation (SD) and minimum and maximum values are presented for mortality rate (%/deck/day), environmental measurements and feed and water consumption for fourteen voyages (Table 3). Daily deck mortality rate averaged 0.048%. The mean deck dry bulb temperature was, on average, one degree hotter, at 29.8 °C, and humidity was higher, at 77%, on the sheep decks than on the bridge. The mean daily water and feed consumption were respectively 5.2 L and 1.3 kg per head.

### 3.1. Bridge and Deck Temperatures

**Figure 1:**
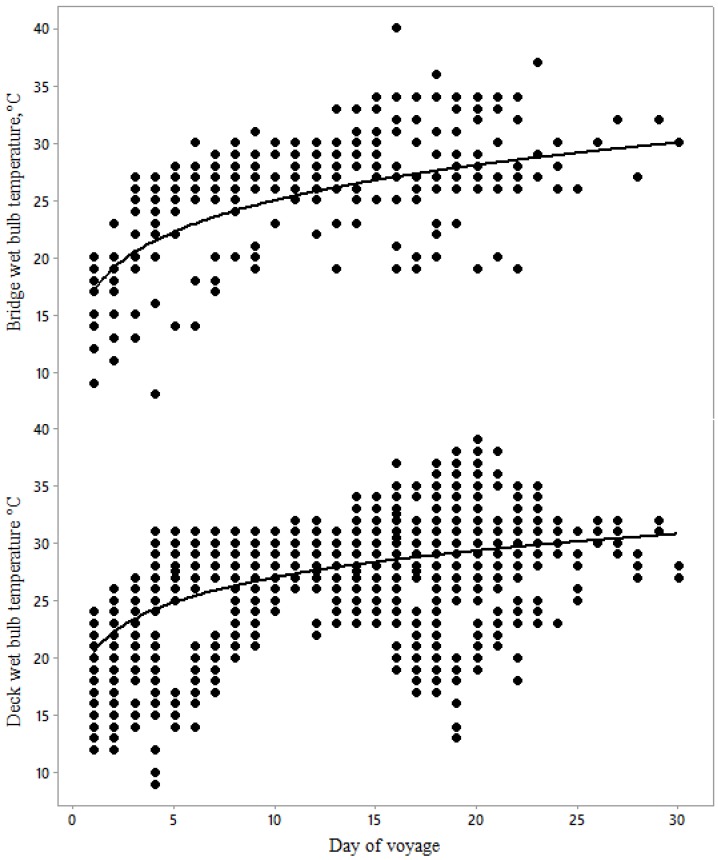
Changes in bridge T_WB_ (°C) and deck T_WB_ during days of all 14 voyages, described by the following equations: Bridge T_WB_, °C = 17.05 (±0.16) × Day^0.17(+0.004)^, F = 17.79 and *p* < 0.0001; Deck T_WB_, °C = 20.55(±0.19) × Day^0.12(+0.004)^, F = 13.34 and *p* < 0.0001, (*n* = 3533).

**Figure 2:**
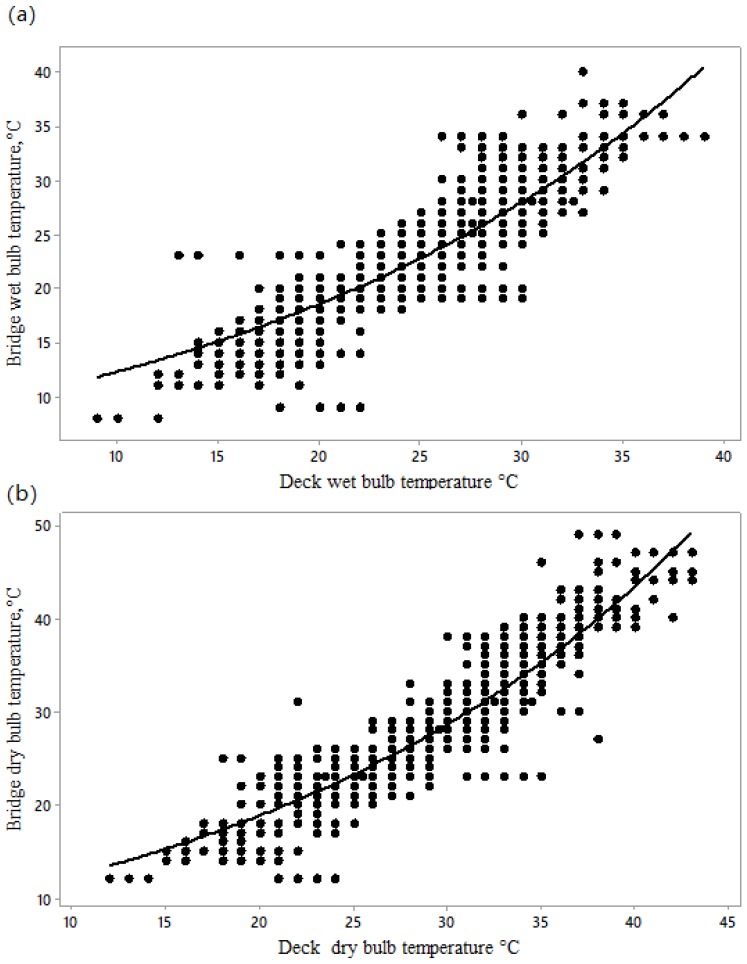
Non-linear exponential models for the relationship between T_WB_ (**a**) and T_DB_ (**b**) on sheep decks and on the bridge, described by the following equations: Bridge T_WB_ = 8.18 (±0.11) exp (0.041 (±0.0005) × Deck T_WB_), F = 9.09 and *p* < 0.0001; Bridge T_DB_ = 8.21 (±0.105) exp (0.042 (±0.0004) × Deck T_DB_), F = 5.59 and *p* < 0.0001, where all temperatures are in °C (*n* = 2681).

Both bridge and deck wet bulb temperature increased over the course of the voyage, more in the early stages of the voyage (Figure 1). As bridge wet and dry bulb temperatures increased, the corresponding deck temperatures increased but less rapidly (Figure 2), probably due to the thermal inertia of the ship decks compared to the relatively exposed bridge.

### 3.2. Mortality Rates

The general linear model identified the variables influencing deck daily mortality rate (%/day) during the 14 journeys (Table 4). Visual inspection of the residuals provided evidence of an approximately normal distribution. In total, 86.8% (R^2^) of the variation was explained by the variables in the model. Mortality increased with day of voyage, as shown in the following power function equation:

Mortality rate (%/deck/d) = 0.0123 (±0.00292) day^0.574(±0.0862)^, F = 2.64, *p* < 0.0001

**Figure 3:**
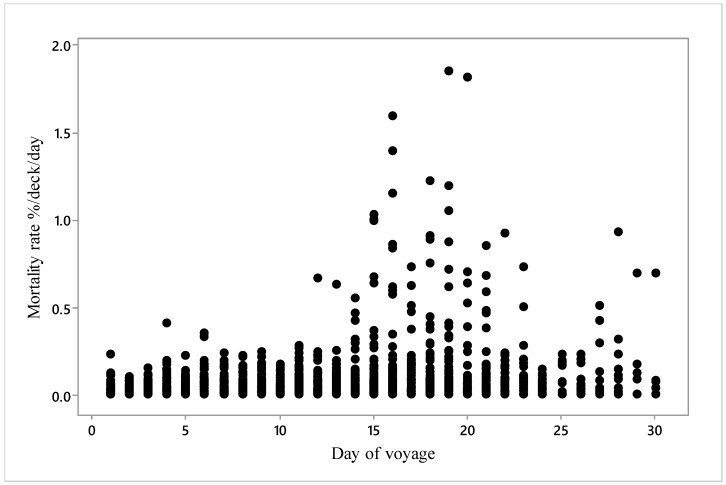
Mortality rate (%/deck/day) during the days of all 14 voyages (*n* = 3026).

Inspection of the relationship demonstrated that the small number of high mortality events were mainly between days 14 and 23 (Figure 3).

**Figure 4:**
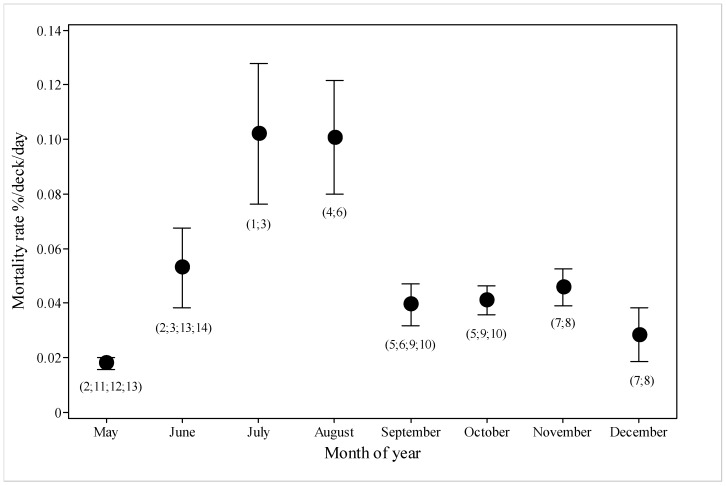
Mortality rate (%/deck/day) for the different months of the voyages (*n* = 14, voyage numbers in parentheses) departing from May to December. Several voyages spanned two months.

Mortality rate (Figure 4) increased during journeys that were made in June compared with May, and then increased further in July and August, before declining again for September to December.

**Figure 5:**
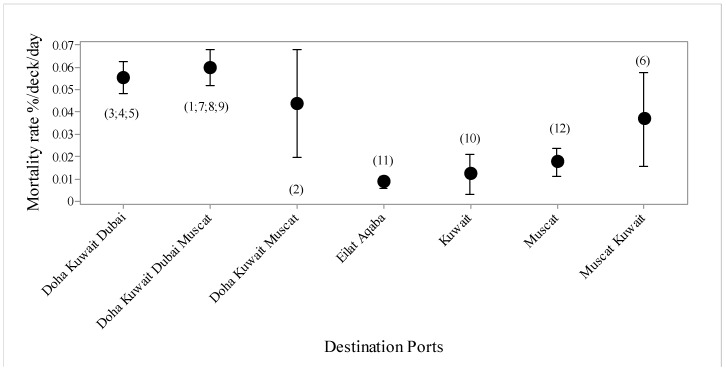
Mortality rate (%/deck/day with standard deviations) for voyages (*n* = 12, voyage numbers in parentheses) offloading at different ports.

**Figure 6:**
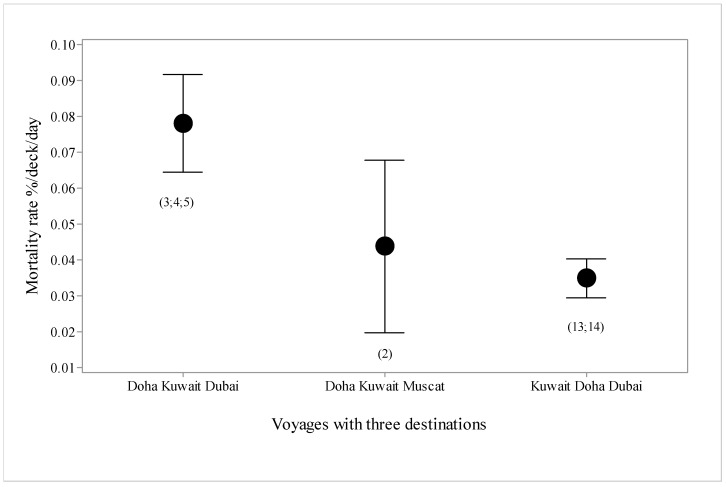
Mortality rate (%/deck/day with standard deviations) for voyages (*n* = 6, voyage numbers in parentheses) with three destination ports in different orders.

**Figure 7:**
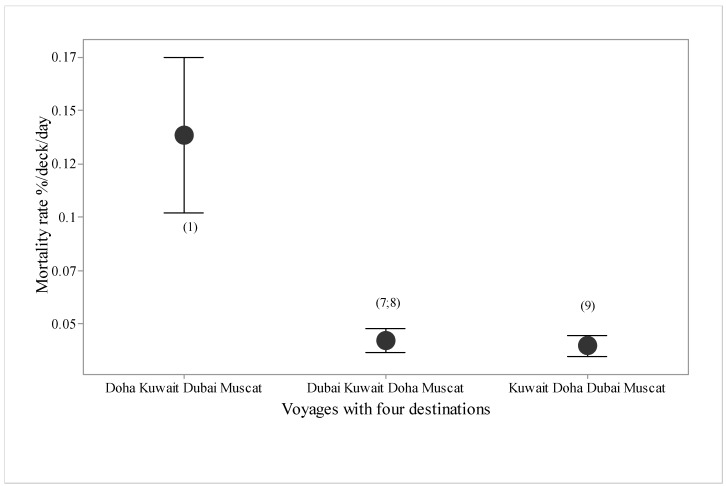
Mortality rate (%/deck/day with standard deviations) for voyages (*n* = 4, numbers in parentheses) with four destination ports in different orders.

There were seven types of voyages, in terms of destination ports (Figure 5), with higher mortality in voyages offloading at Doha, Kuwait and Dubai, and these three plus Muscat, compared with Kuwait, Muscat or Eilat alone. Mean duration in port was 1.55 days. Voyages to Muscat tended to have higher mortality than voyages to Eilat. For the three destination voyages, mortality rate was over twice as high for voyages offloading at Doha, then Kuwait and then Dubai, compared with those offloading at Kuwait, then Doha and then Dubai (Figure 6). For the four destination voyages, mortality was almost three times as high for vessels offloading at Doha, then Kuwait, then Dubai then Muscat than if Doha was either the second or third destination in the voyage (Figure 7).

**Figure 8:**
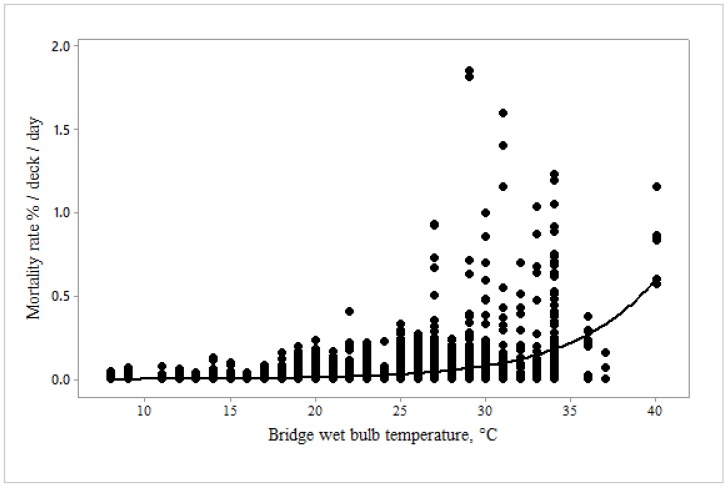
Mortality rate (%/deck/d) changes with bridge wet bulb temperature (°C) (*n* = 2798).

**Figure 9:**
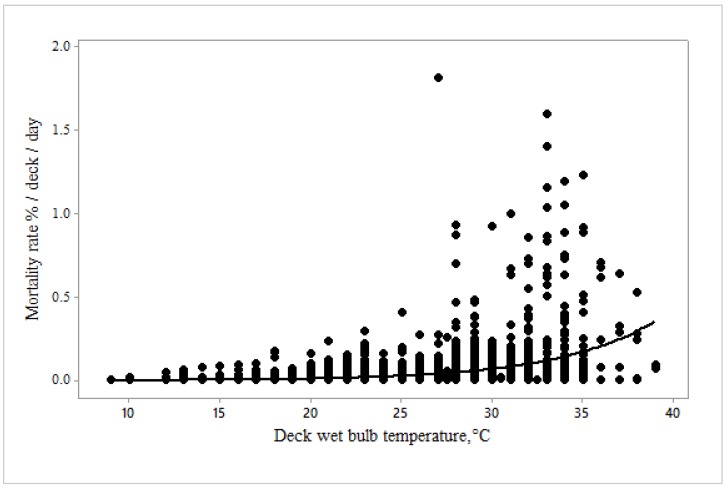
Mortality rate changes with deck wet bulb temperature °C (*n* = 2638).

Mortality rate increased exponentially with bridge (Figure 8) and deck (Figure 9) wet bulb temperatures. At high temperatures it went up more gradually for the deck compared with the bridge.

The relationship between mortality rate %/deck/day and bridge T_WB_ was described by the equation:

Mortality rate (%/deck/d) = 0.0002 (±0.00004) × exp 0.2 (±0.007) Bridge T_WB_, °C, F = 7.66, *p* < 0.0001

The relationship between mortality rate %/day and deck T_WB_ was described by the equation:

Mortality rate %/deck/day = 0.0003 (±0.000009) × exp 0.18 (±0.0095) Deck T_WB_, F = 8.85, *p* < 0.0001.

**Figure 10:**
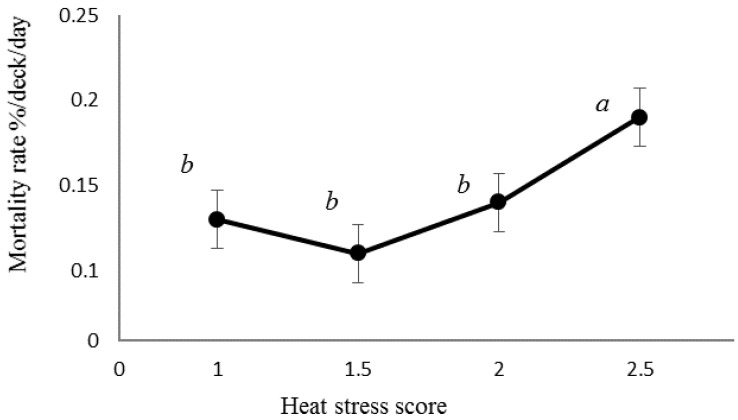
Mortality rates for heat stress character scores (with standard errors of the mean values) between 1 and 2.5. No scores of 3 were awarded. 1 = normal, 2 = moderate and 3 = severe. Means with different superscripts were significantly different (*p* < 0.05) by Fisher’s exact test.

Mortality rates (%/deck/day) were significantly increased for sheep in heat stress score 2.5, i.e., moderate to severe, compared with lower scores (Figure 10).

**Figure 11:**
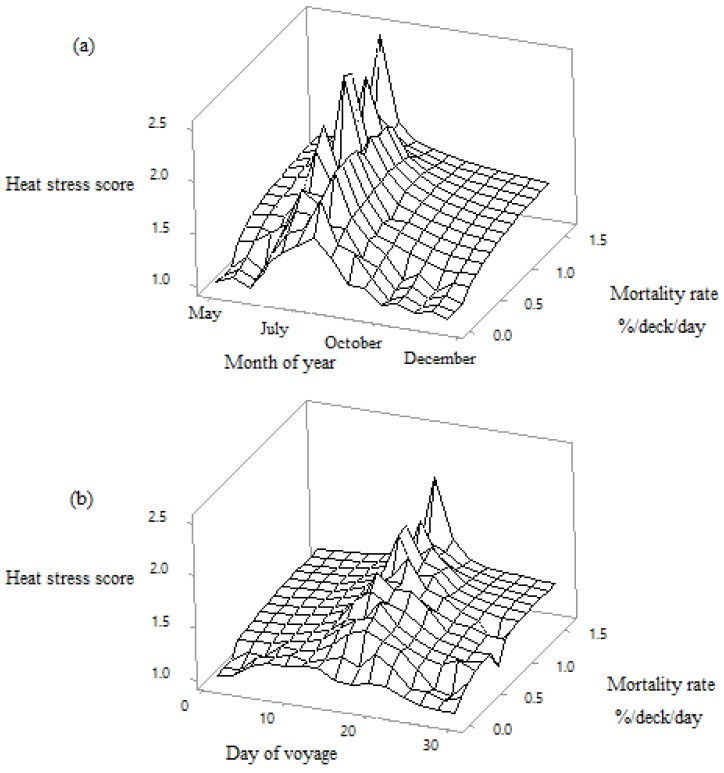
Relationships between heat stress score (1 = normal, 2 = moderate and 3 = severe), mortality and both month (**a**) and day (**b**) of the voyage.

The increase in mortality in the month of July was associated with a high heat stress score, and this occurred between day 15 and 20 of the voyage (Figure 11). This was when the ships arrived at the first port. The five journeys that had Doha as the first destination had on average 0.13% ± 0.08 SD mortality when at the first port, during which time a mean of 58.35% ± 12.49 SD of the total sheep were offloaded. The nine voyages that had other first destinations only had on average 0.03% + 0.02 SD mortality.

**Figure 12:**
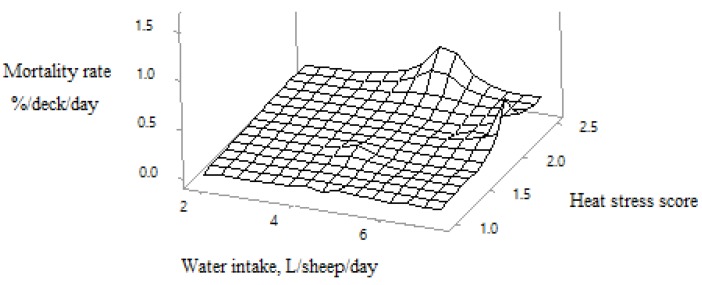
Relationships between water consumption, mortality and heat stress score (1 = normal, 2 = moderate and 3 = severe).

**Figure 13:**
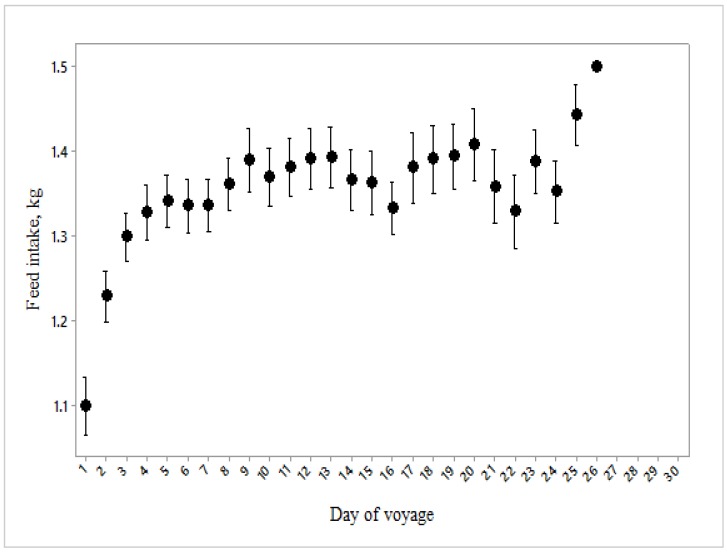
Feed consumption per sheep over the 27 days of the voyage (*n* = 3784).

**Figure 14:**
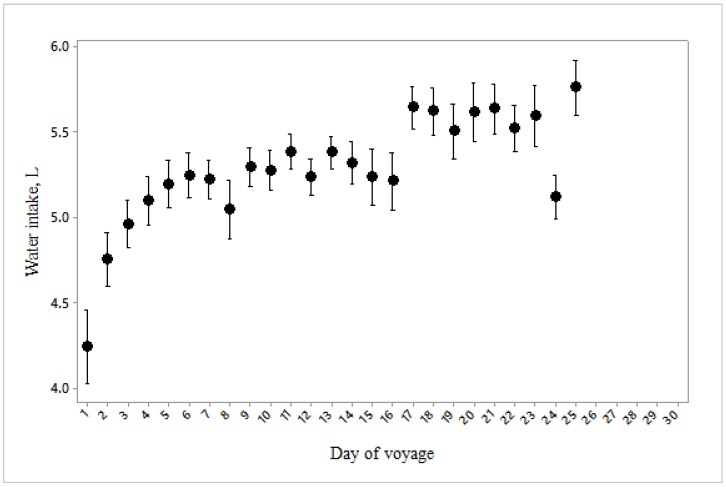
Water consumption per sheep during the 25 days of the voyage (*n* = 3581).

Mortality rate was also increased when a mean water intake of approximately 5.5 L/d combined with high heat stress scores, but not at higher water intakes (Figure 12). Feed and water intakes increased curvilinearly over the duration of the voyage (Figure 13 and Figure 14).

#### Parameters Related to Heat Stress Scores

**Figure 15:**
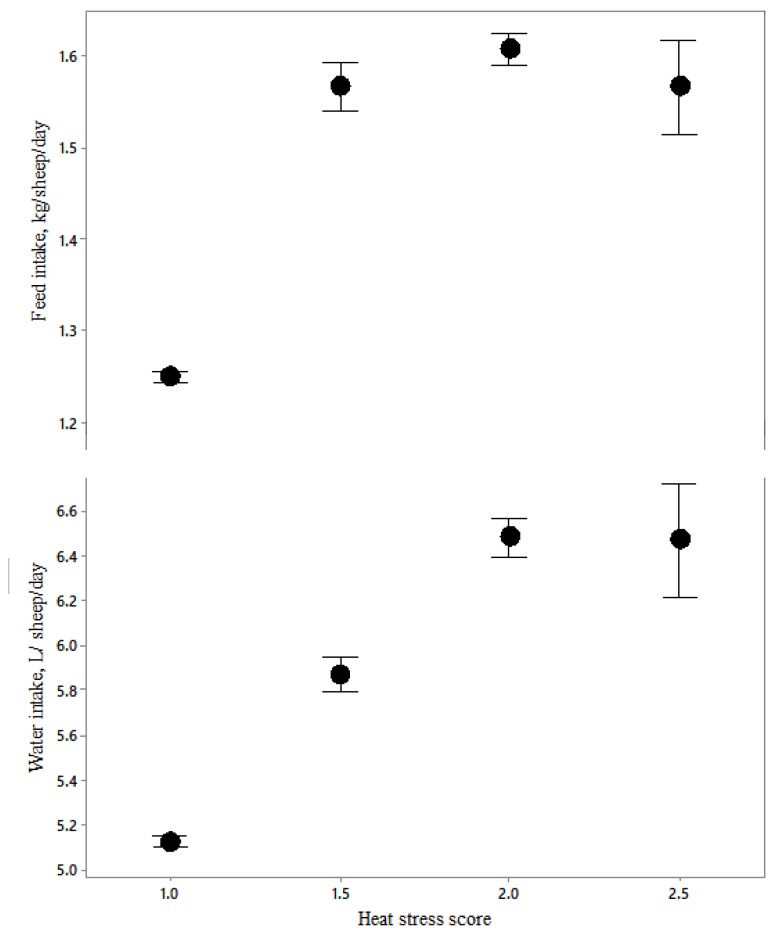
The relationships between feed and water consumption and heat stress score (*n* = 3022).

**Figure 16:**
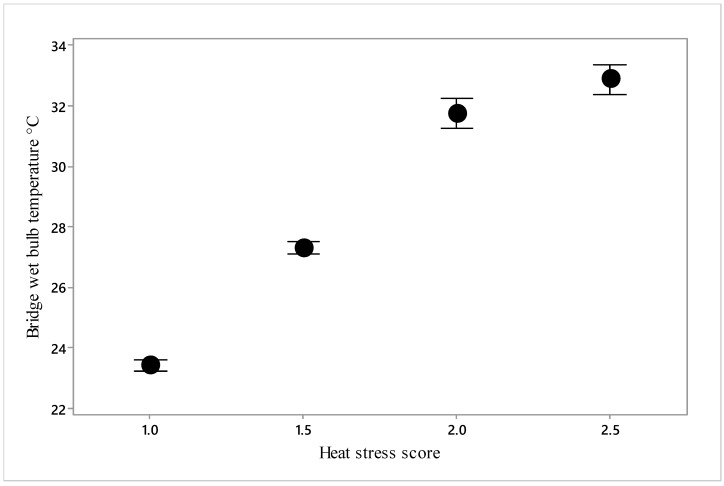
The relationship between bridge T_WB_ and heat stress score (1 = normal, 2 = moderate and 3 = severe) (*n* = 3023).

Using ordinal logistic regression, it was determined that four parameters had significant relationships to heat stress scores (Table 5): feed and water intake, bridge T_WB_, and vessel humidity and respiratory character score. These relationships are depicted in Figure 15 and Figure 16.

As heat stress score increased above 1, feed consumption increased, but there was no further intake at higher heat stress scores (Figure 15). Water consumption increased from scores 1.0 to 1.5 to 2.0 but increased no further at 2.5. Heat stress score also increased in a curvilinear relationship with bridge T_WB_ (Figure 16).

## 4. Discussion

Our objective was to determine critical thresholds for reduced welfare at high temperatures, thereby facilitating improved control of the heat stress risk. The relationship between heat stress score and bridge wet bulb temperature determined that an increase in heat stress score to 1.5, i.e., between normal and moderate levels, occurred when bridge T_WB_ reached 27.3 °C (Figure 16), a score of 2 being reached at 31.7 °C. At these scores, mortality rate was not significantly increased according to the model (Figure 10); however, the point of inflection of the curve in Figure 8 demonstrates that mortality begins to increase somewhere between 25 and 30 °C bridge T_WB_. Thus, welfare can reasonably be said to be impaired, as some heat stress has been recognised and there is limited evidence of increased mortality.

The mean 50th and 98th percentile values for average wet bulb temperatures in July to September have been measured in Kuwait (26.7 and 31.8 °C, respectively), Qatar, Doha (27.5 and 32.2 °C, respectively), United Arab Emirates, Dubai (27.7 and 31.5 °C, respectively), and Oman, Muscat (26.2 and 29.8 °C, respectively) [13]. Thus, if it is assumed that bridge T_WB_ reflects temperature in these ports, there is a 50% chance that voyages offloading at Doha or Dubai between the months of July and September will cause an elevation of heat stress score. Voyages offloading at Kuwait and Oman present a slightly reduced risk. Our data also showed that bridge T_WB_ increased from 25 °C to 30 °C from day 10 to the end of transport (Figure 1).

The Australian Government draft report [7] recommends certain limitations for deck T_WB_, which can be related to bridge T_WB_ using our equation T_WB_ = 8.18 exp (0.041 Deck T_WB_). The report defines the heat stress threshold for sheep as the maximum ambient T_WB_ at which heat balance of the deep body temperature can be controlled using available mechanisms of heat loss [22]. The rate of change of ambient temperature may influence the temperature at which the core body temperature of sheep begins to increase, since ship mortality records suggest that sudden increases are associated with a rapid rise in sheep mortality [10]. The heat stress threshold is the same as the upper critical temperature defined by Brody [23], above which physical regulatory mechanisms, principally cutaneous and respiratory evaporative heat loss in this case, can no longer prevent core body temperature increasing.

The research confirms increased mortality in July and August (Figure 4), reported in previous studies [8,24]. Reducing travel during the Australian winter period and concentrating it into autumn may also decrease sheep mortality but would not match key demand periods in the Middle East.

The study showed a correlation between destination ports and mortality rate. The first port destination was reached on average after 16.3 days (Section 2.1) and the highest mortality was recorded for voyages offloading at Doha as the first port for animal unloading (Figure 5). Thus, ships should offload to their furthest destinations first, which will have lower mortality rates, and only offload at Doha, a high-risk port, last.

Mortality was high when heat stress increased, accompanied by increased water intake. Water intake was not measured in this study and the values were allocations. As well as the invidious effects of heat stress per se, increasing temperature also increases ammonia volatilization, which is a further source of discomfort to the sheep [5].

### Limitations

The first limitation of this study is that, due to a lack of details for the environmental monitoring on the ships, we assume the relevant data were accurately recorded and were representative of temperature and humidity on sheep decks or on the bridge of the ship. For instance, recording sea water temperature from the intake ports has inherent variance, because these ports vary in depth by several metres, depending on the ship [25], and are usually deeper for livestock ships (15 ± 8.6 m) than other merchant vessels [26]. Engine intake temperature measurements are typically about 0.3 °C greater than direct measurements [27]. T_WB_ on sheep decks might be under-reported by several degrees because only a single daily measurement for T_WB_ is made on each deck by ship veterinarians, not necessarily at the hottest time of the day [12], which modelling work has shown to be insufficient sampling [27]. Furthermore, the temperature on the ship is not the same in all areas within a deck, with high temperatures generally occurring beside the engine block, boiler room and heated fuel tank [14]. We also related bridge temperatures to those in the port, which should be accurate whilst the ship is in the port, but at sea the humidity is likely to be higher and therefore heat stress risk increased. In the port, the cross ventilation of open decks is limited, compared with out at sea, potentially increasing the mortality rate. Doubt also surrounds the tautologous water intake data, since these were allocated according to temperature, and correlations with temperature were inevitable. As water use by the sheep was not measured on a regular basis, and values were based on the veterinarian’s allocation, there is obvious potential for error, due to spillage or inaccurate allocation. Accurate measures of water intake would be valuable, particularly to monitor the sheep’s response to heat stress, given that water is available ad libitum. Regarding the scale used for heat stress, there are many available, but this one was tailored to behaviour likely to be exhibited by sheep on ships. It is currently not validated, but the correlations observed in this study will go some way in this process.

In contrast to the modelling results based on 417 voyages from Australia to the Middle East [8], this study did not identify any increased mortality risk on shipments leaving Australia in the Southern Hemisphere spring, which was probably due to the limited voyages during this time of year that we included in our study.

## 5. Conclusions

Heat stress is first evident at temperatures of approximately 27.5 °C T_WB_ at the bridge. Approximately 50% of voyages offloading at Doha and Dubai will experience these temperatures. Mortality is particularly increased during offloading at Doha, compared to other ports, if this is the first port of call. As mortality is increased during the winter season of the Southern Hemisphere, this suggests a change to increase travel at other times of year and avoid Doha as the first stop.

## Figures and Tables

**Table 1:** The world’s countries in rank order of sheep population, and numbers of animals exported and imported (×10^6^) [2].

Sheep Population		No. Exported/Year × 10^6^		No. Imported/Year × 10^6^	
China	162.1	Sudan	4.84	Saudi Arabia	7.17
Australia	67.5	Romania	2.55	Kuwait	1.18
India	64.3	Somalia	2.16	Italy	1.1
Nigeria	42.1	Australia	1.87	Greece	0.54
Iran	41.8	Spain	1.13	Qatar	0.53
Sudan	40.5	Hungary	0.55	Oman	0.40
UK	33.9	France	0.48	Yemen	0.38
Ethiopia	30.6	Mauritania	0.20	France	0.26
Pakistan	29.8	Saudi Arabia	0.18	Lebanon	0.16

**Table 2:** Physical indicators of heat stress in sheep in the three-point scale used by ship veterinarians to score sheep daily on each deck.

Indicator	Normal	Moderate	Severe
Respiration rate, breaths/min	15–70	70–160	>160
Panting	None	Visible panting, some open mouth	Shallow panting, all open mouth
Distress	None	Some	Severe
Drooling	None	Some	White, sticky thick foam
Eating	Normal	Reduced	Unable to eat, choking and no interest in food
Drinking	Normal	Small regular drinks	Cannot drink without choking. Loss of interest in drinking
Neck position	Relaxed	Extended to improve airflow	Extended towards passageways, head over water trough
Movement	Normal	Reduced	Reluctant to move, crowding around railings/vents/smothering/climbing on each other
Stance	Normal	Front legs splayed	Front legs splayed sideways; front legs also extended forwards and back legs backwards

**Table 3:** Descriptive statistics for sheep mortality rate (%/deck/day), environmental measurements and feed and water intake from fourteen voyages from Australia to the Middle East.

Variable ^1^	No. Records	Mean	SD	Minimum	Maximum
Mortality rate %/deck/day	3026	0.048	0.12	0.00	1.84
Deck T_DB_, °C	2728	29.8	4.73	12.0	43.0
Deck T_WB_, °C	2724	26.7	4.63	9.0	39.0
Deck RH, %	2724	77.4	10.85	23.0	93.0
Deck THI	2724	28.8	4.46	4.5	41.0
Bridge T_DB_, °C	3534	28.8	6.03	12.0	49.0
Bridge T_WB_, °C	3534	24.8	5.08	8.0	40.0
Bridge Relative humidity, %	3534	71.9	12.58	15.0	92.0
Bridge THI	3534	27.5	5.22	12.2	41.5
Feed intake, kg/sheep/day	3581	1.34	0.22	0.8	2.0
Water intake, L/sheep/day	3573	5.22	0.91	1.63	7.2

^1^ T_DB_ = Dry Bulb Temperature, T_WB_ = Wet Bulb Temperature, RH = Relative Humidity, THI = Temperature-Humidity Index.

**Table 4:** Significant (*p* < 0.05) variables related to sheep mortality rate (%/deck/day) in a general linear model with R^2^ (adj) = 85.14%.

Variable	F-Value	*p*-Value
Day of voyage	23.22	<0.0001
Sheep number, head/deck	17.19	<0.0001
Deck T_WB_, °C	22.08	<0.0001
Water intake, L/head/day	20.31	<0.0001
Exporter	2.67	0.014
Faeces score	41.06	<0.0001
Day of voyagex sheep number	18.53	<0.0001
Day of voyage × Deck T_WB_	23.89	<0.0001
Day of voyage × Water intake	22.17	<0.0001
Sheep number × Deck T_WB_	16.02	<0.0001
Sheep number × Water intake	13.96	<0.0001
Deck T_WB_ × Water intake	19.50	<0.0001
Day of voyage × Exporter	7.01	<0.0001
Day of voyage × Deck number	6.13	<0.0001

**Table 5:** Variables significantly (*p* < 0.05) related to heat stress score (1 = normal, 2 = moderate and 3 = severe) by ordinal logistic regression: with data from 14 voyages and 22–30 days for each voyage.

Heat Stress Score	Odds Ratio	% 95 CI		Coef	*p*-Value
		Lower	Upper		
Water intake, L/sheep/day	0.37	0.18	0.75	−0.997216	0.005
Feed intake, kg/sheep/day	0.00	0.00	0.00	−8.10772	<0.0001
Bridge T_WB_, °C	0.31	0.14	0.72	−1.15803	0.006
Vessel humidity, %	1.18	1.03	1.37	0.169587	0.021

## Appendix A

**Table A1:** Summary table of information relating to the 14 trips studied; duration of voyages, specifying the various ports and the time spent unloading sheep with an average of 1.55 days.

Voyages N°	N° Tot Sheep (Head)	Departure Load Port	Duration (d) N° Total Stops	First Stop N°day Data Location-Days in Port	Second Stop N°day Data Location-Day in Port	Third Stop N°day Data Location-Day in Port	Fourth Stop N°day Data Location-Day in Port
1	69,322	04/07/16 Fremantle	25 4	d 14 17/07/16 Doha-2 d	d 18 21/07/16 Kuwait-1 d	d 20 23/07/16 Dubai-1 d	d 22 25/07/16 Muscat-2 d
2	44,999	15/05/17 Fremantle	22 3	d 15 29/05/17 Doha-2 d	d 19 02/06/17 Kuwait-3 d	d 22 05/06/17 Muscat 1 d	-
3	63,038	22/06/17 Fremantle	23 3	d 16 07/07/17 Doha-3 d	d 19 10/07/17 Kuwait-3 d	d 23 14/07/17 Dubai-1 d	-
4	63,804	02/08/17 Fremantle	24 3	d 15 16/08/17 Doha-1 d	d 17 18/08/17 Kuwait-4 d	d 21 22/08/17 Dubai-3 d	-
5	59,873	14/09/17 Fremantle	20 3	d 15 28/09/17 Doha-2 d	d 18 01/10/17 Kuwait-1 d	d 20 03/10/17 Dubai-1 d	-
6	17,917	15/08/17 Adelaide	25 2	d 19 02/09/17 Muscat-3 d	d 23 06/09/17 Kuwait-2 d	-	-
7	77,988	14/11/17 Fremantle	22 4	d 15 28/11/17 Dubai-4 d	d 20 03/12/17 Kuwait-1 d	d 21 04/12/17 Doha-1 d	d 22 05/12/17 Muscat 1 d
8	6669	14/11/17 Fremantle	23 4	d 15 28/11/17 Dubai-1 d	d 17 30/11/17 Kuwait-4 d	d 21 04/12/17 Doha-2 d	d 23 06/12/17 Muscat 1 d
9	73,591	26/09/17 Fremantle	24 4	d 17 12/10/17 Kuwait-3 d	d 20 15/10/17 Doha-3 d	d 22 17/10/1 Duba-2 d	d 24 19/10/17 Muscat 1 d
10	4466	26/09/17 Fremantle	17 1	d 17 12/10/17 Kuwait-2 d	-	-	-
11	39,390	04/05/18 Fremantle	24 1	d 19 22/05/18 Eilat-2 d	d 21 24/05/18 Aqaba -1d		-
12	15,325	04/05/18 Fremantle	13 3	d 13 16/05/18 Muscat 1d	-	-	-
13	57,208	10/05/18 Fremantle	30 3	d 24 02/06/18 Kuwait-3 d	d 27 05/06/18 Doha-2 d	d 29 07/06/18 Dubai-1 d	-
14	57,428	Fremantle 07/06/18	19 4	d 14 20/06/18 Kuwait-3 d	d 17 23/06/18 Doha-2 d	d 19 25/06/18 Dubai 1 d	-
