# Correction: Carnovale, F.; Phillips, C.J.C. The Effects of Heat Stress on Sheep Welfare during Live Export Voyages from Australia to the Middle East. *Animals* 2020, *10*, 694

**DOI:** 10.3390/ani11082297

**Published:** 2021-08-04

**Authors:** Francesca Carnovale, Clive J. C. Phillips

**Affiliations:** 1 Centre for Animal Welfare and Ethics, School of Veterinary Science, The University of Queensland, Gatton, QLD 4343, Australia; francesca.carnovale@student.emu.ee; 2 Institute of Veterinary Medicine and Animal Sciences, Estonian University of Life Sciences, Kreutzwaldi 1, 51014 Tartu, Estonia

The authors wish to make the following correction to this paper [1]:

On page 3, first paragraph, the third line should read 46,499 (SD 25,459), not 46,501 (SD 25,461), and the 13th line should read 22.21 ± 4.06, not 22.36 ± 4.13. On page 9, paragraph 1, line 3, mean duration in port should be 1.95 d, not 1.55 d. We apologize for any inconvenience. The authors wish to change the Appendix Table A1. The new table is as follows:

In the title, average duration has been changed from 1.55 to 1.95 days, in column 2, voyage 5 has been changed from 59,873 to 59,823; voyage 7 has been changed from 77,988 to 77,998; in column 3 voyage 6 has been changed from 2017 to 2016, Fremantle has been changed to Adelaide-Fremantle and voyage 13 from Fremantle to Adelaide and the date need to be moved up in voyage 14; in column 4 number of stops for voyages 11, 12 and 14 have been changed from 1, 3 and 4 to 2, 1 and 3, respectively; in column 5 and 6 voyage 6 have been changed from 2017 to 2016.

## Figures and Tables

**Table A1:** Summary table of information relating to the 14 trips studied; duration of voyages, specifying the various ports and the time spent unloading sheep with an average of 1.95 days.

Voyages N°	N° Tot Sheep (Head)	Departure Load Port	Duration (d) N° Total Stops	First Stop N°day Data Location-Days in Port	Second Stop N°day Data Location-Day in Port	Third Stop N°day Data Location-Day in Port	Fourth Stop N°day Data Location-Day in Port
1	69,322	04/07/16 Fremantle	25 4	d 14 17/07/16 Doha-2 d	d 18 21/07/16 Kuwait-1 d	d 20 23/07/16 Dubai-1 d	d 22 25/07/16 Muscat-2 d
2	44,999	15/05/17 Fremantle	22 3	d 15 29/05/17 Doha-2 d	d 19 02/06/17 Kuwait-3 d	d 22 05/06/17 Muscat 1 d	-
3	63,038	22/06/17 Fremantle	23 3	d 16 07/07/17 Doha-3 d	d 19 10/07/17 Kuwait-3 d	d 23 14/07/17 Dubai-1 d	-
4	63,804	02/08/17 Fremantle	24 3	d 15 16/08/17 Doha-1 d	d 17 18/08/17 Kuwait-4 d	d 21 22/08/17 Dubai-3 d	-
5	59,823	14/09/17 Fremantle	20 3	d 15 28/09/17 Doha-2 d	d 18 01/10/17 Kuwait-1 d	d 20 03/10/17 Dubai-1 d	-
6	17,917	15/08/16 Adelaide-Fremantle	25 2	d 19 02/09/16 Muscat-3 d	d 23 06/09/16 Kuwait-2 d	-	-
7	77,998	14/11/17 Fremantle	22 4	d 15 28/11/17 Dubai-4 d	d 20 03/12/17 Kuwait-1 d	d 21 04/12/17 Doha-1 d	d 22 05/12/17 Muscat-1 d
8	6669	14/11/17 Fremantle	23 4	d 15 28/11/17 Dubai-1 d	d 17 30/11/17 Kuwait-4 d	d 21 04/12/17 Doha-2 d	d 23 06/12/17 Muscat-1 d
9	73,591	26/09/17 Fremantle	24 4	d 17 12/10/17 Kuwait-3 d	d 20 15/10/17 Doha-3 d	d 22 17/10/1 Duba-2 d	d 24 19/10/17 Muscat-1 d
10	4466	26/09/17 Fremantle	17 1	d 17 12/10/17 Kuwait-2 d	-	-	-
11	39,390	04/05/18 Fremantle	24 2	d 19 22/05/18 Eilat-2 d	d 21 24/05/18 Aqaba-1 d		-
12	15,325	04/05/18 Fremantle	13 1	d 13 16/05/18 Muscat-1 d	-	-	-
13	57,208	10/05/18 Adelaide	30 3	d 24 02/06/18 Kuwait-3 d	d 27 05/06/18 Doha-2 d	d 29 07/06/18 Dubai-1 d	-
14	57,428	07/06/18 Fremantle	19 3	d 14 20/06/18 Kuwait-3 d	d 17 23/06/18 Doha-2 d	d 19 25/06/18 Dubai-1 d	-
